# BK polyomavirus: latency, reactivation, diseases and tumorigenesis

**DOI:** 10.3389/fcimb.2023.1263983

**Published:** 2023-09-13

**Authors:** Xianfeng Zhou, Chunlong Zhu, Hui Li

**Affiliations:** ^1^ Cancer Research Center, Jiangxi University of Chinese Medicine, Nanchang, China; ^2^ Jiangxi Engineering Research Center for Translational Cancer Technology, Nanchang, China; ^3^ Jiangxi Provincial Health Commission Key Laboratory of Pathogenic Diagnosis and Genomics of Emerging Infectious Diseases, Nanchang Center for Disease Control and Prevention, Nanchang, China; ^4^ Clinical Laboratory, Third Hospital of Nanchang, Nanchang, China

**Keywords:** BK polyomavirus, latency, BKV-associated nephropathy (BKVAN), reactivation, tumorigenesis

## Abstract

The identification of the first human polyomavirus BK (BKV) has been over half century, The previous epidemiological and phylogenetic studies suggest that BKV prevailed and co-evolved with humans, leading to high seroprevalence all over the world. In general, BKV stays latent and symptomless reactivation in healthy individuals. BKV has been mainly interlinked with BKV-associated nephropathy (BKVAN) in kidney-transplant recipients and hemorrhagic cystitis (HC) in hematopoietic stem cell transplant recipients (HSCTRs). However, the mechanisms underlying BKV latency and reactivation are not fully understood and lack of extensive debate. As Merkel cell polyomavirus (MCV) was identified as a pathogenic agent of malignant cutaneous cancer Merkel cell carcinoma (MCC) since 2008, linking BKV to tumorigenesis of urologic tumors raised concerns in the scientific community. In this review, we mainly focus on advances of mechanisms of BKV latency and reactivation, and BKV-associated diseases or tumorigenesis with systematical review of formerly published papers following the PRISMA guidelines. The potential tumorigenesis of BKV in two major types of cancers, head and neck cancer and urologic cancer, was systematically updated and discussed in depth. Besides, BKV may also play an infectious role contributing to HIV-associated salivary gland disease (HIVSGD) presentation. As more evidence indicates the key role of BKV in potential tumorigenesis, it is important to pay more attention on its etiology and pathogenicity *in vitro* and *in vivo*.

## 1 Introduction

BK polyomavirus (BKV), the first human polyomavirus isolated from an immunosuppressed kidney transplant recipient in 1971, is a member of the *Polyomaviridae* family of double-stranded DNA (dsDNA) viruses. “BK” is named after the initials of this patient (Gardner et al., 1971). In the same year, JC polyomavirus (JCV) was identified from specimens of brain pathology of a patient diagnosed of progressive multifocal leukoencephalopathy (PML) (Padgett et al., 1971). Identification of the first two viruses accelerated understanding of the pathogenicity of human polyomaviruses. For more than three decades, the BKV and JCV were the only well-known polyomaviruses associated with clinical diseases in specific groups of people. With the technological progress of modern molecular methods and next-generation sequencing technique, 13 polyomaviruses were included in the list of human polyomaviruses during the past 20 years (Prezioso et al., 2021) (Zhou et al., 2019). The previous epidemiological and phylogenetic studies suggest that BKV prevailed and co-evolved with humans, leading to its high seroprevalence all over the world (Viscidi et al., 2011; Gaboriaud et al., 2018; Zhou et al., 2019; Zhou et al., 2020). Based on genome sequence diversity, BKV has be divided into six genotypes, in which genotype I is considered as the most frequent worldwide (~80%), followed by genotype IV (15%) (Kotla et al., 2021). BKV is ubiquitous around the globe, with up to 90% of adults being seropositive, and the transmission routes of BKV were speculated through direct contact or fecal-oral transmission during childhood (Krajewski et al., 2020; Furmaga et al., 2021).

In general, BKV infection is self-limited and then remains latent in the urinary tissue for life time among immunocompetent individuals (Furmaga et al., 2021). Nevertheless, in some immunocompromised patients, BKV can reactivate with high level of viral replication. Most commonly, BKV reactivation leads to BKV-associated nephropathy (BKVAN) in some kidney transplant recipients (KTRs). And in some allogeneic hematopoietic stem cell transplant recipients (HSCTRs), the consequences of viral reactivation may be hemorrhagic cystitis (HC) (Krajewski et al., 2020; Laskin et al., 2020). However, the molecular mechanisms of BKV latency and pathogenicity after reactivation are not comprehensively discussed. In addition, BKV was also considered as a potential factor or co-factor of tumorigenesis (Burger-Calderon and Webster-Cyriaque, 2015) (Saber Amoli et al., 2021). And accumulated evidence links BKV to urinary tumors such as prostate and bladder cancer (Mischitelli et al., 2015; Vaezjalali et al., 2018; Villani et al., 2019). In addition, BKV was also linked to HIV-associated salivary gland disease (HIVSGD) in HIV-infected individuals, and HIVSGD is associated with increased lymphoma incidence (Burger-Calderon et al., 2016).

This review will focus on BKV latency, reactivation and the associated diseases, especially concentrate studies on organ-transplant recipients (OTRs), among whom viral reactivation might cause fatal damage. Moreover, the latest studies on the relationship between BKV and different types of cancers will be addressed and discussed.

## 2 BKV epidemiology

In general, primary BKV infection occurs during childhood, as studies have shown that 60%-70% of children were seropositive of anti-BKV IgG by the age of 10 (Krajewski et al., 2020; Furmaga et al., 2021). BKV infection is considered to be transmitted through direct human-to-human contact or fecal-oral route, and respiratory route was also speculated to contribute to the high seroprevalence (Furmaga et al., 2021). As most BKV infections are asymptomatic and self-limited, it is not possible to confirm these transmission routes. Serological studies support that primary exposure to BKV occurs during early childhood, and then stay latent in most adult (Viscidi et al., 2011; Prelog et al., 2013; Sroller et al., 2014). The anti-BKV seroprevalence is low in children at their first 6 months with the gradual weakening of protection from maternal antibodies and reaches to 80%-90% among adults worldwide (Viscidi et al., 2011). In the past decades, as the establishment of virus-like particle-based ELISA and multiplex immunoassays, plus new members of human PyVs were constantly identified, more attention was paid to human PyVs and seroprevalence investigations on these viruses were conducted in many countries (**Supplementary Table 1**) (Stolt et al., 2003; Kean et al., 2009; Nicol et al., 2013; Prelog et al., 2013; Sroller et al., 2014; Zhang et al., 2014; Fukumoto et al., 2015; Laskin et al., 2015; Gaboriaud et al., 2018; Kamminga et al., 2018; Laine et al., 2023). Overall, most sero-epidemiology studies were conducted in developed countries in which BKV, and JCV were the most concerned pathogens. Whereas limited seroprevalence data was from low-income countries, indicating PyV-associated diseases were relatively neglected in the developing world.

## 3 Mechanisms of BKV latency

**Figure 1:**
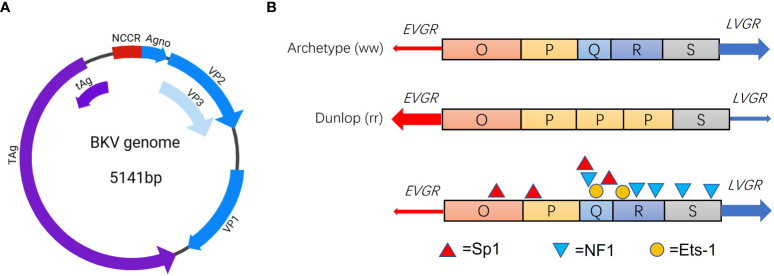
Genome structure of BKV **(A)** and NCCR blocks of the archetype and Dunlop strain **(B)**. Major TFBS Sp1 (red triangle), NF1 (blue triangle) and Ets-1 (orange circle) in the NCCR blocks of BKV.

In general, BKV, after primary infection, sustains a persistent latent stage in epithelial cells of renal tubules or urothelium for life time under normal conditions (McCaffrey et al., 2021). BKV entry into host cells is mediated via caveolae, entry into the cell is then driven by a caveola-mediated endocytic pathway (Eash et al., 2004). After entering the nucleus, the BKV genome remains episomal in human cells (Gorrill et al., 2006). In recent years, latency mechanism behind viral genome of BKV that enables its coexistence with human hosts aroused more concerns. BKV is a small (diameter 40 nm) non-enveloped icosahedral virus (Furmaga et al., 2021). BKV genome is a double-stranded DNA of approximately 5,000 base pairs (bp) long and comprises three major regions, early viral gene region (EVGR), late viral gene region (LVGR) and noncoding control region (NCCR). The EVGR codes for early T proteins, small t antigen (tAg) and large T antigen (TAg), and LVGR codes for viral capsid proteins VP1, VP2, VP3 and agnoprotein (Agno) (**Figure 1A**) (Blackard et al., 2020). Based on sequence variation of VP1 gene, BKV has been universally classified into four genotypes (genotype I- IV) (Vaezjalali et al., 2018). As reported, genotype I of BKV are the most frequent around the globe (80%), while genotype IV is mainly reported from countries of Europe and northeastern Asia (Chen et al., 2006) (Hu et al., 2018). In addition to genotyping based on BKV VP1 diversity, two other forms based on NCCR variations were universally accepted for clinical isolates, namely, archetype (ww) and rearranged (rr) variants such as Dunlop strain (**Figure 1B**) (Henriksen et al., 2015). The BKV archetype contains a 376 bp linear OPQRS block, in which O represents the start of replication and PQRS represents promoters and regulatory regions of EVGR and LVGR (Kotla et al., 2021) (Bethge et al., 2016). Whereas the rearranged variants of BKV occur due to deletion and duplication in the NCCR sequences during reactivation and persistent replication (**Figure 1B**).

On the LVGR, BKV encodes one precursor miRNA complementary to the 3′ coding end of the TAg mRNA (Seo et al., 2008). Broekema and Imperiale found that miRNA plays a key role in limiting replication of archetype BKV by targeting viral early mRNA in an infection model using renal proximal tubule epithelial cells (RPTE), suggesting a self-limiting replication mechanism to remain life-time latency (Broekema and Imperiale, 2013). However, in rearranged NCCR (rr-NCCR) variants, as the miRNA expressed in a low level, early mRNA are expressed in high levels with enhanced early promoter activity (Broekema and Imperiale, 2013). In addition, innate and adaptive immune regulation on virus-host interaction also play an important role for BKV to sustain persistent latency in humans. For instance, the viral miRNA BKV-miR-B1-3p can target the stress-induced ligand ULBP3, a protein recognized by the receptor natural killer group 2, member D (NKG2D). Consequently, BKV-miR-B1-3p downregulated expression level of ULBP3 to evade NKG2D recognition, which leads to NKG2D-mediated elimination (Bauman et al., 2011; Zeng et al., 2019). This immune regulation mechanism of virus-host interaction has been extensively accepted. Recently, miRNA of BKV was clinically used to monitor viral reactivation in blood and urine of KTRs (Demey et al., 2021; Demey et al., 2022).

Besides, a recent study found BKV agnoprotein is able to impair innate immune signaling by disrupting the mitochondrial network and then enhances mitophagy. Specifically, BKV agnoprotein impairs IRF3 nuclear translocation and induces mitochondrial fragmentation. Then the disrupted mitochondria are targeted for SQSTM1/p62 (an autophagy receptor) mitophagy and impair innate immune signaling (Manzetti et al., 2020). Interestingly, a few studies showed the agnoprotein were able to co-localize with lipid‐droplets (LD) *in vitro* and the predicted α‐helical region ranging from amino acids 22 to 42 of BKV agnoprotein is vital for the localization (Unterstab et al., 2010). Besides, BKV agnoprotein has potentially been involved in disrupting exocytosis (Johannessen et al., 2011), inhibiting viral replication (Gerits et al., 2015) and facilitating the virus egress (Panou et al., 2018).

Previous studies indicated that small t antigen (tAg) of polyomaviruses involves in important pathways regulating viral replication, the innate immune signaling, and transformation for SV40, JCV and Merkel cell polyomavirus (MCV) (Chen et al., 2007; Saribas et al., 2019). However, the regulation mechanism underlying BKV tAg has been less focused. Zou and Imperiale found that BKV tAg downregulated viral DNA replication through similar mechanism of SV40 tAg replacing the B’ regulatory subunit of protein phosphatase 2A (PP2A) to form a complex to promote cell cycle progression (Zou and Imperiale, 2023). More studies elucidating molecular mechanisms of tAg in regulating viral replication are needed.

Recently, Zhao and Imperiale established a novel cell model mimicking viral latency and activation of BKV using a human RPTE cell line expressing human telomerase reverse transcriptase (RPTE-hTERT) (Zhao and Imperiale, 2021). They found that the archetype BKV can persist *in vitro* for ~100 days with random recombination checked by single-molecule high-throughput sequencing. Eventually, the accumulated recombination events could lead to rr-NCCR that allows higher efficiency of BKV DNA replication (Zhao and Imperiale, 2021). This study provides a useful *in vitro* model for future studies of viral persistence and reactivation.

**Figure 2:**
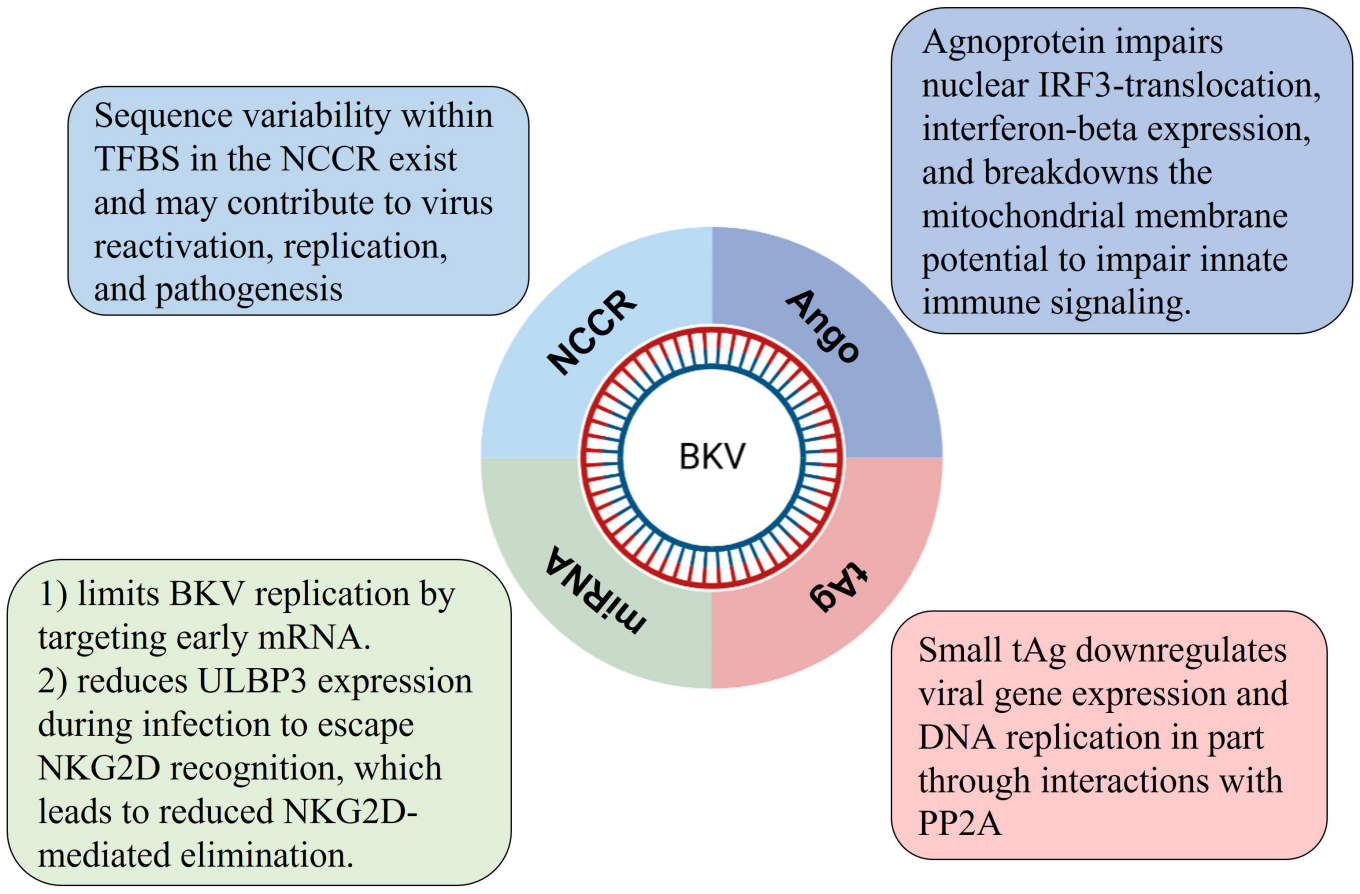
Four possible regulating mechanisms of BKV latency regulated by viral genes.

Taken together, there are several potential molecular or immune mechanisms connected to BKV latency in association with NCCR, miRNA, Agno and tAg regulation as summarized in **Figure 2**.

## 4 BKV reactivation and its potential mechanisms

Latent infection of BKV might last for life-time for most immunocompetent individuals. However, BVK predominantly reactivates and causes diseases in immunocompromised population, particularly the kidney transplant recipients (KTRs), hematopoietic stem cell transplantation (HSCT) recipients and HIV/AIDS patients (Nankivell et al., 2017; Pan et al., 2018; Hirsch and Randhawa, 2019; Raupp et al., 2019; Wunderink et al., 2019) (Laskin et al., 2020). Interestingly, over 80% of immunocompetent adults are seropositive for BKV as previously reported (**Supplementary Table 1**), but BKVAN occurs almost exclusively in KTRs, which raises concerns on the underlying pathogenesis mechanisms and attracts mounting concern as number of worldwide kidney transplants increases. BKVAN is clinically confirmed by tissue biopsy, and then clinical management through immunosuppression modulation is essential to balance exacerbation of the disease and acute rejection (Chantziantoniou et al., 2016). It was reported that about 60% of KTRs have detectable BK viruria, and up to 10% of the patients develop BKVAN (Babel et al., 2011; Chen et al., 2020). 15%-50% of the BKVAN patients will progress to graft loss within 2-3 years in the absence of proper intervention (Leeaphorn et al., 2020). Evidence indicated that the development of BKVAN was linked to immunosuppressive regimens for KTRs (Leeaphorn et al., 2020; Cheung and Tang, 2022). It has been found that patients taking more powerful immunosuppressive drugs, such as mycophenolate and tacrolimus, are more likely to develop BKVAN (Mehrvar et al., 2021). Therefore, frequent monitoring on viral load of BKV in the first year after transplantation, and timely adjustments or rational reduction of immunosuppression are highly recommended (Dalianis et al., 2019). However, reduction of immunosuppression using immunosuppressant drugs has a double-edged sword effect on clinical outcomes with increasing risk of acute rejection (Cheung and Tang, 2022).

Generally, high viral load of BKV (>10^4^ copies/ml plasma or >10^7^ copies/ml urine) are considered as indications of viral reactivation (Marinic et al., 2014; Ambalathingal et al., 2017). With reactivation, BKV potentially disrupt cell-cycle regulation and innate immune response, then significantly increase the level of viral replication, leading to cell necrosis and flaking. The exfoliated cells with viral inclusions in urine specimens are termed “decoy cells” as they are easily misdiagnosed as cancer cells (Chantziantoniou et al., 2016). Decoy cells (DC) that can be detected in the fresh urine sediment using microscopy are used to prompt the early signs of BKV activation (Poloni et al., 2016). Although DC can be identified on urine cytology, but it’s positive predictive value for BKVAN diagnosis is low (Chen et al., 2020) (Hirsch et al., 2002). Moreover, many of KTRs with positive urinary DC did not develop BKVAN (Huang et al., 2013). Therefore, optimizing diagnostic methods of accurate identification of BKV-infected DC is valuable for clinical diagnosis and treatment decisions.

It is widely recognized that both rr-NCCR of BKV and immunosuppression of the host jointly promote the development of BKVAN. There are many kinds of transcription factor binding sites (TFBS) in NCCR of archetype BKV, sequence variability of the TFBS may contribute to viral activation, replication, and pathogenesis (Blackard et al., 2020). In order to compare functional differences of rr-NCCR, Olsen et al. reconstructed Dunlop strain of BKV by replacing the NCCR from 12 BKV isolates of urine or renal biopsy specimen in Vero cells, and observed impressive difference of replication efficiency in RPTEs, indicating that sequence variability of NCCR has impact on replication efficiency of BKV (Olsen et al., 2009).

BKV strains exhibit higher-level genetic diversity in the NCCR than that in protein coding regions. Forms of rr-NCCR are commonly identified from persons with BKV-associated diseases (Cubitt, 2006). It is found that BKV isolates with rr-NCCR replicate much more efficiently than archetype BKV, indicating NCCR rearrangements regulate bidirectional gene expression levels (Gosert et al., 2008). An *in vitro* study finds that deletion or duplication in different blocks of NCCR may lead to elevated or decreased viral genome replication, indicating that rearrangements of NCCR contribute to regulate viral protein expression (Helle et al., 2017). The rr-NCCR caused by block deletion or duplication will alter the distribution and composition of TFBS, which in turn regulates the viral gene expression (Bethge et al., 2016) (Bethge et al., 2015). Bethge et al. identified Sp1 site in NCCR as a key regulator of gene expression of EVGR and LVGR (Bethge et al., 2015). And the *in vitro* experiments suggest that transcription factors Ets1, NF-1 and Sp1 (**Figure 1B**) determine the strength toward early or late gene expression (Bethge et al., 2015). This study provides new evidence on how different composition of TFBS regulate early and late gene expression of BKV and contribute to viral replication. Therefore, the potential anti-viral therapeutic strategies based on specific transcription factors will be promising for patients.

## 5 BKV-associated diseases and potential tumorigenesis

High seroprevalence of human polyomaviruses indicate its ubiquity in nature. The major human tissues harboring BKV are in urinary system such as the kidney and bladder (Krajewski et al., 2020). It is a complex network in pathogen-host interactions, which cause the diverse clinical outcomes of BKV infection in humans (Babel et al., 2011) (Nankivell et al., 2017). Anyway, BKV-associated diseases were mainly linked to host immune dysfunction in transplant recipients taking immunosuppressant drugs, patients undergoing cancer treatment and HIV/AIDS patients with immunodeficiency (Broekema et al., 2010; Mitterhofer et al., 2014). The most common clinical outcome due to BKV reactivation in transplant recipients are BKVAN, which can lead to graft loss in up to 60% of affected patients (Babel et al., 2011). Other than that, BKV was also considered as a potential factor or co-factor of tumorigenesis and diseases that were much less discussed in the scientific community. Among the known human oncogenic viruses, human papillomavirus (HPV) has similar genomic structure with BKV, offering an excellent reference model to understand the potential mechanisms of BKV-induced tumorigenesis (Papadimitriou et al., 2016). Similar with HPVs, BKV, as its key tumorigenesis mechanism, has an TAg-mediated disruption of the tumor suppressor genes p53 and pRb, which consequently leads to dysregulation of cell cycling and apoptosis (Trave and Zanier, 2016) (Storey et al., 1998; Narisawa-Saito and Kiyono, 2007).

### 5.1 BKV-associated urologic tumors

BKV builds persistent latent infection in the genitourinary system of humans for life time (McCaffrey et al., 2021). As a member of human polyomaviruses, BKV shares similar genome structures with oncogenic virus MCV that causes a malignant cutaneous cancer Merkel cell carcinoma (MCC) (Helle et al., 2017). BKV was linked as a potential etiological agent of urologic diseases or tumors such as prostate and bladder cancer. As development of high throughput sequencing, studies with the deep sequencing technology have recently begun to understand the frequency and potential networks of BKV-associated tumorigenesis (Starrett and Buck, 2019). Two former studies of comprehensive molecular characterization of bladder cancers on the basis of deep sequencing technique observed that gene of BKV was integrated into the genome in 1 of 413 bladder tumors (Robertson et al., 2017) (Cancer Genome Atlas Research N, 2014). The findings suggest low incidence of BKV gene integration into bladder tumor genome in the immunocompetent individuals. However, recent observations have revealed that KTRs who develop BKV viremia or BKVAN have about 11-fold risk of bladder cancer incidence in comparison to KTRs without signs of BKV reactivation (Liu et al., 2017) (Gupta et al., 2018). These findings specifically implicate that BKV reactivation is the precondition of bladder cancerogenesis in immunosuppressed transplant recipients.

To our knowledge, prostate cancer is a popular urinary tumor in the elderly of developed countries and disrupted p53 networks is thought to be the major pathways for prostate cancer incidence (Mischitelli et al., 2015). TAg of BKV is responsible for viral transformation, evidence suggests that molecular mechanisms of BKV tumorigenesis is linked to TAg-mediated p53 inhibition (Harris et al., 1996). Recently, a case-control study conducted by Gorish and colleagues observed that BKV TAg was identified among 30% (n=55) tissue specimens of prostate cancer patients but only in 7% (n=55) of the controls’ specimens (*P*=0.002 and Odd ratio= 5.7), suggesting that BKV is a potently associated with higher risk of prostate cancer (Gorish et al., 2019).

### 5.2 BKV-associated head and neck cancer

Head and neck cancers are a heterogeneous group of tumors representing the 6^th^-7^th^ most popular types of cancers around the world (Mody et al., 2021). Approximately 90% of head and neck cancers belong to squamous cell carcinomas (HNSCCs). And the activated virus infections are one of major causative agents (Polz et al., 2015) (Kitamura et al., 2023).

A recent study assessed the prevalence of BKV in Iranian patients with brain malignancies, and found TAg sequences of BKV were detected in 26 out of 58 (44.8%) brain tumor tissues, indicating the possible pathogenic interlink between BKV persistence and central nervous system (Saber Amoli et al., 2021). Another study investigated the correlation of BKV and the development of papillary thyroid carcinoma (PTC) in Iranian PTC patients. Among 1057 PTC samples including 645 paraffin-embedded and 412 fresh biopsy samples, 48.3% were positive for the BKV DNA with mean viral load of 0.5×10^4^ copies/cell. Besides, TAg RNA expression was relatively higher in fresh biopsy samples (Tarharoudi et al., 2022). Polz and colleague analyzed the presence of BKV in paraffin-embedded sections of oral squamous cell carcinomas (OSCC), and they observed that BKV nucleic acid was detected in 18.5% (n=92) of OSCC patients but much lower detection rate (3.3%) from the controls (Polz et al., 2015). However, reports of BKV-associated head and neck cancer are still too limited to determine its etiological interlink with head and neck cancer. After all, BKV is ubiquitous and life-long latent in humans.

### 5.3 BKV association in HIV-associated salivary gland disease

After primary infection, BKV mainly disseminates and predominantly colonizes into sites of kidney and urinary tracts (Imperiale, 2000). Interestingly, BKV DNA was also detected from saliva of HIV-infected individuals and HIV-negative controls, which raised concerns of its pathogenicity in people living with HIV (Jeffers and Webster-Cyriaque, 2011). HIV-associated salivary gland disease (HIVSGD) is one of the most common salivary gland-associated complications among HIV-infected population (Jeffers and Webster-Cyriaque, 2011; Burger-Calderon et al., 2016). Generally, HIVSGD presents xerostomia or/and diffused swelling. The incidence of HIVSGD could reach up to 48% in HIV-positive individuals in underdeveloped countries (McArthur et al., 2000). According to Patton’s data observed during 1995-2008, HIV/AIDS patients were much more likely to develop HIVSGD in the era of protease inhibitor therapy (Patton et al., 2000). Although HIVSGD is generally thought to be a benign lesion, some of them could progress to malignant lymphoma under certain conditions like HIV infection (Ellis, 2007). Notably, lymphomas account for a large portion of major salivary gland malignancies, in which salivary gland lymphomas occur in about 75–80% parotid gland, 5–20% submandibular gland, and less than 5% small sublingual salivary glands (Barnes et al., 1998). With accumulated evidence interlinking head and neck cancers with BKV, question about whether BKV may contribute as co-factorial role to tumorigenesis.

Since Jeffers et al. detected significantly higher BKV viral loads in the saliva of patients diagnosed with HIVSGD as compared to HIV negative patients, evidence linking BKV to HIVSGD has augmented (Jeffers et al., 2009). The BKV NCCR rearrangement derived from block duplications and/or deletions commonly occurs in immunocompromised patients with BKV reactivation. Burger-Calderon et al. found over 90% of the BKV NCCRs in HIVSGD carried a block arrangement form “OPQPQQS” in throatwash samples of the immunosuppressed individuals (Burger-Calderon et al., 2016). It has been reported that rearrangements of NCCR potently enhanced viral transformation and host-cell permissiveness (Gosert et al., 2008) (Burger-Calderon et al., 2016). Taken together, BKV may play an infectious role contributing to HIVSGD presentation. However, future studies will have to address the pathogenicity of BKV *in vitro* and *in vivo* specifically.

## 6 Conclusions

BKV is a ubiquitous agent causing latent infection in over 80% adults around the world. Most concern on this virus is BKVAN in KTRs, among whom up to 60% eventually progress to graft loss in the first 2-3 years in the absence of proper intervention. Thus, clinical recommendations on management of BKV infection include: a) frequently monitoring of viral load of BKV in blood is highly recommended the first 12 months after kidney transplantation; b) in order to the risk for BKVAN incidence, reducing the use of immunosuppressants is recommended (Dalianis et al., 2019). Generally, the BKV has a self-limiting regulating mechanism in association with miRNA that targets early mRNA to limit archetype BKV replication. Theoretically, BKV is latent in archetype among most immunocompetent individuals unless there is a sustained weakening of the immune system caused by HIV infection or kidney transplant. The activation of BKV is usually accompanied by block rearrangement of NCCR in which TFBS deletion or insertion will regulate EVGR and LVGR expression. Therefore, NCCR sequence could be an important indicator to evaluate the BKV activation. Although human RPTE cell are the major harboring sites for its life-long latency, BKV has tropism for normal human brain tissue, human salivary gland cells and pancreatic cells *in vitro*, which indicates BKV association with various tumors in non-genitourinary tissues (Elsner and Dorries, 1992; Jeffers et al., 2009).

Approximately 12% of human cancers are related to viruses such as Epstein-Barr virus (EBV), human papillomavirus (HPV), hepatitis B virus (HBV), hepatitis C virus (HCV), Kaposi’s sarcoma herpesvirus (KSHV) and MCV (White et al., 2014). The accumulated evidence in the past two decades suggests that BKV may cause urologic tumors and head and neck cancers as well, posing new challenges to the immunosuppressed individuals in the absence specific anti-viral drugs or vaccines. Altogether, figuring out the pathogenic mechanisms causing BKV-associated diseases and potential tumorigenesis is important, and the constant improvement of rapid clinical diagnosis for BKV activation is needed.

## Author contributions

XZ: Conceptualization, Supervision, Writing – original draft, Writing – review and editing. CZ: Data curation, Investigation, Resources, Writing – review and editing. HL: Data curation, Investigation, Resources, Writing – review and editing.
