# Supplementary material for: BK polyomavirus: latency, reactivation, diseases and tumorigenesis
Source: Front Cell Infect Microbiol. 2023 Sep 13;13:1263983. doi: 10.3389/fcimb.2023.1263983 (PMC10525381; doi:10.3389/fcimb.2023.1263983)
Supplement: Supplementary file 1 [file Table_1.docx]

Supplementary Table 1 List of representative seroepidemiological studies of human PyVs around the world (1975-2023)

| **Country** | **Sample size** | **Population** | **Age** | **BKV (%)** | **Other PyVs** | **Methods** | **Year of publication** | **References (DOI)** |
| --- | --- | --- | --- | --- | --- | --- | --- | --- |
| Finland | 459 | Pregnant women and spouses | NS | 95-99 | JCV | GST-capture ELISA | 2023 | 10.1038/s41598-023-27850-7 |
| France | 551 | Renal transplant patients and healthy control | NS | 76.7-88.4 | / | VLP-based ELISA | 2021 | 10.1093/ndt/gfab279 |
| China | 1620 | HIV/ADIS patients and healthy controls | 18-80 | 71.6-76.2 | JCV, MCV, TSV, NJV | VLP-based ELISA | 2020 | 10.1038/s41598-020-74244-0 |
| Netherlands | 152 | Healthy blood donors | NS | NS | HPyVs 2-14 | multiplex bead-based immunoassay | 2018 | 10.1128/JCM.01566-17 |
| USA | 36 | Children prior to allogeneic hematopoietic cell transplant | 5-15 | 100 | / | VP1 capsid | 2015 | 10.1002/pbc.25536 |
| Japan |  | Healthy individuals | 1-70 | 1050 | JCV, MCV | VLP-based ELISA | 2015 | 10.1371/journal.pone.0115646. |
| Czech Republic | 991 | Healthy individuals | 6-64 | 69 | JCV, MCV | VLP-based ELISA | 2014 | 10.1002/jmv.23841 |
| Germany | 50 | Thymectomized patients and healthy controls | 2-5 | 65.6-70.6 | JCV | VLP-based ELISA | 2013 | 10.1016/j.jcv.2013.08.035 |
| Spain | 2117 | Bladder cancer and control | NS | 60-64 | JCV, MCV | VLP-based ELISA | 2013 | 10.1002/ijc.28053 |
| Italy | 947 | Outpatient | 1-93 | 54.9-78.9 | JCV, MCV | VLP-based ELISA | 2011 | 10.1128/CVI.05175-11 |
| Australia | 458 | Corhort of skin cancer | 25-60 | 94-99 | JCV | Luminex platform | 2010 | 10.1099/vir.0.020115-0 |
| Switzerland | 400 | blood donors | 20-59 | 71-87 | JCV | VLP-based ELISA | 2009 | 10.1086/597126. |
| USA | 1501 | Healthy pediatric population and adult blood donors | 1-70 | 82 | JCV, MCV, KIV, WUV, LPV | recombinant VP1 capsid proteins | 2009 | 10.1371/journal.ppat.1000363 |
| Uganda | 821 | Patients of different cancer | NS | 86.3 | / | BK virus | 2006 | 10.1097/00008469-200608000-00002 |
| Sweden | 1033 | Pregnant women and controls | NS | 80 | / | VLP-based ELISA | 2005 | 10.1002/ijc.20573 |
| USA | 1346 | Non-Hodgkin lymphoma cases and control | 20-74 | 57-79 | JCV | VP1 proteins | 2005 | 10.1002/ijc.21277 |
| Sweden | 590 | Health children | 1-13 | 20-98 | JCV | VLP-based ELISA | 2003 | 10.1099/vir.0.18842-0 |
| UK | 2435 | General population | 5-69 | 81 | JCV | HI antibody | 2003 | 10.1002/jmv.10450 |
| Japan | 732 | healthy volunteers and in-patients | 20-100 | 54.5-63.1 | JCV | EIA (VP1) | 1990 | 10.11150/kansenshogakuzasshi1970.64.1507 |
| New Hebrides and Solomon Islands | 1544 | 28 isolated populations | NS | 0-89 | JCV, SV40 | HI antibody | 1975 | 10.1093/oxfordjournals.aje.a112169 |

NS: not stated
